# Double Burden of Malnutrition in Rural West Java: Household-Level Analysis for Father-Child and Mother-Child Pairs and the Association with Dietary Intake

**DOI:** 10.3390/nu7105399

**Published:** 2015-10-02

**Authors:** Makiko Sekiyama, Hong Wei Jiang, Budhi Gunawan, Linda Dewanti, Ryo Honda, Hana Shimizu-Furusawa, Oekan S. Abdoellah, Chiho Watanabe

**Affiliations:** 1 Graduate Program in Sustainability Science-Global Leadership Initiative (GPSS-GLI), Graduate School of Frontier Sciences, The University of Tokyo, 5-1-5 Kashiwanoha, Kashiwa City 277-8563, Japan; 2 Research Institute for Humanity and Nature, 457-4 Motoyama, Kamigamo, Kita-ku, Kyoto 603-8047, Japan; jiang@chikyu.ac.jp; 3 Institute of Ecology, Research Institute, Padjadjaran University, Jl. Sekeloa Selatan I, Bandung 40132, Indonesia; budhi_gunawan@unpad.ac.id (B.G.); oekan.abdoellah54@gmail.com (O.S.A.); 4 Faculty of Medicine, Airlangga University, Jl. Mayjen. Prof. Dr. Moestopo 47, Surabaya 60132, Indonesia; lindaperisdiono@yahoo.com; 5 RSET, Institute of Science and Engineering, Kanazawa University, Kakuma-machi, Kanazawa 920-1192, Japan; rhonda@se.kanazawa-u.ac.jp; 6 Department of Human Ecology, School of International Health, The University of Tokyo, 7-3-1 Hongo, Bunkyo-ku, Tokyo 113-0033, Japan; hana-shimizu@umin.ac.jp (H.S.-F.); chiho@humeco.m.u-tokyo.ac.jp (C.W.)

**Keywords:** double burden, malnutrition, adiposity, food frequency questionnaire, Indonesia

## Abstract

Indonesia is facing household-level double burden malnutrition. This study aimed at examining (1) household-level double burden for the mother-child and father-child pairs; (2) risk of adiposity of double burden households; and (3) associated dietary factors. Subjects were 5th and 6th grade elementary school children (*n* = 242), their mothers (*n* = 242), and their fathers (*n* = 225) in five communities (1 = urban, 4 = rural) in the Bandung District. Questionnaires on socioeconomic factors, blood hemoglobin measurements, and anthropometric measurements were administered. For adults, body fat percentage (BF%) was estimated by bioelectrical impedance (BF%-BI) and by converting skinfold thickness (ST) data using Durnin and Womersley’s (1974) formula (BF%-ST). Food frequency questionnaires were also completed. Double burden was defined as coexistence of maternal or paternal overweight (Body mass index (BMI) ≥ 23) and child stunting (height-for-age z-score <−2) within households. Maternal-child double burden occurred in 30.6% of total households, whereas paternal-child double burden was only in 8.4%. Mothers from double burden households showed high adiposity; 87.3% with BF%-BI and 66.2% with BF%-ST had BF% >35%, and 60.6% had waists >80 cm. The major dietary patterns identified were “Modern” and “High-animal products”. After controlling for confounding factors, children in the highest quartile of the “High-animal products” dietary pattern had a lower risk of maternal-child double burden (Adjusted OR: 0.46, 95% CI: 0.21–1.04) than those in the lowest quartile. Given that the “High-animal products” dietary pattern was associated with the decreased risk of maternal-child double burden through a strong negative correlation with child stunting, improving child stunting through adequate intake of animal products is critical to solve the problem of maternal-child double burden in Indonesia.

## 1. Introduction

Nutrition transition comprises food consumption and physical activity changes caused by lifestyle transformations resulting from rapid urbanization and modernization [1]. Whereas this process occurred gradually in developed countries, in many developing countries it has been proceeding at a faster rate [2]. Consequently, many developing countries are facing increasing rates of overweight and obesity [3,4], though undernutrition is still prevalent in these countries. This coexistence of overnutrition and undernutrition is often referred as the double burden of malnutrition [2,5,6]. Researchers have revealed that double burden occurs not only at the country level [5,7], but also at the household level [8,9].

As for household-level double burden, most literature has examined the relationship between mother and child. These studies revealed that in several countries mothers are overweight whereas their children are stunted in the same household [9,10]. However, the relationship between father and child at the household level has scarcely been investigated.

Body mass index (BMI) has been commonly used to define maternal overweight in double burden households [2,6,9,10], despite the fact that it does not directly measure adiposity. Excess adiposity is associated with increased risk of non-communicable diseases (NCDs) such as type 2 diabetes and cardiovascular disease [11,12]. NCDs are the leading cause of global disease burden [13], with 80% of mortality from NCDs occurring in low- and middle-income countries [14]. However, the actual risk of adiposity among mothers in double burden households has not been studied.

Identification of risk factors of the double burden household has been investigated from the perspective of socioeconomic characteristics of these households [10,15,16]. Several studies have revealed that double burden is associated with older maternal age [6,10], maternal short stature [15,16], larger family size [15,17], and higher levels of maternal education [6,10]. However, food consumption patterns of household members, which mediate socioeconomic characteristics of the household, and physical characteristics of household members, have scarcely been studied as associated factors of double burden. For example, higher risk of double burden in urban residents was interpreted to be associated with a rapid shift to inactivity and an energy-dense diet [10], but empirical data for explaining such an association are limited. One such example of the limited literature is that of a study in Malaysia, which concluded that the variety of food available to children decreases the risk of double burden [17].

Until recently, three studies have reported double burden of malnutrition at the household level in Indonesia [2,6,18] using secondary data analysis with a large sample size. The first study analyzed data from the Indonesian Nutrition Surveillance System (INSS) in 2000–2003 and found that overweight mother and stunted child pairs were found among 11% of rural households in Indonesia [6]. The latter two studies used the same dataset, the Indonesian Family Life Survey (IFLS), from different years. Roemling and Qaim [2] analyzed 1993 (IFLS1), 1997 (IFLS2), 2000 (IFLS3), and 2007 (IFLS4) data as a panel and found the proportion of double burden households increased between 1993 and 1997, but remained relatively stable since that time. Vaezghasemi analyzed 2007 (IFLS4) data and found that 19% of households had at least one underweight and one overweight member of the household [18]. These studies have investigated determinants of double burden households using socioeconomic characteristics of the households, whereas the relationship between double burden and food intake patterns has not been analyzed.

The objectives of this study were (1) to examine double burden structure at the household level, not only for the mother-child pair but also for the father-child pair; (2) to compare the adiposity related physical characteristics of double burden household members and non-double burden household members; and (3) to explore the association of dietary patterns of the household members with the occurrence of double burden. The targets for this study were five communities (1 urban and 4 rural) in the Bandung District in West Java, Indonesia.

## 2. Methods

### 2.1. Study Area and Subjects

**Figure 1:**
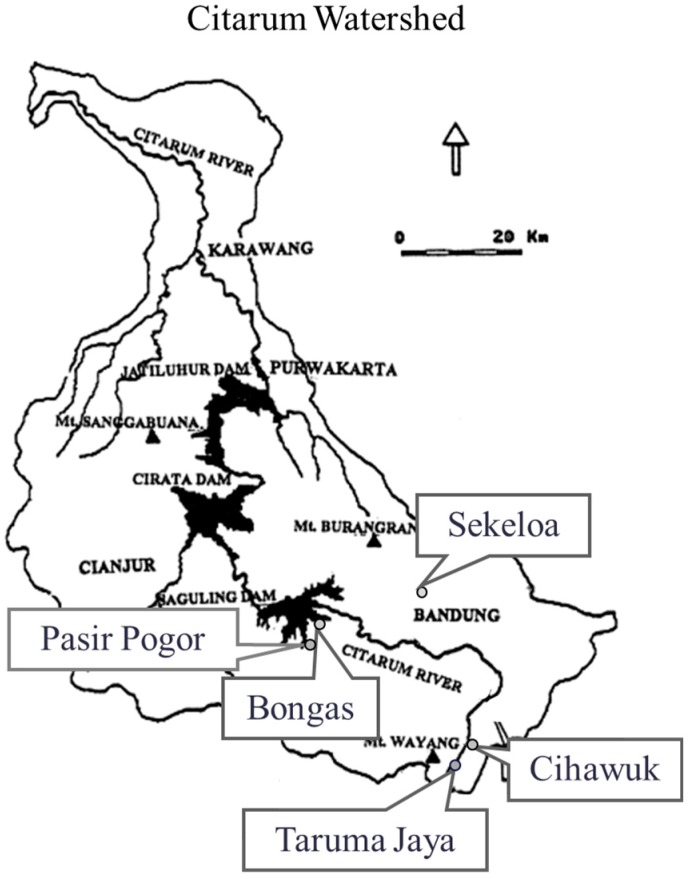
Map of the study sites.

The present study was conducted as a part of the Environmental Research in Rural Asia (ENVRERA) project that aimed to examine the effects of subsistence change (*i.e.*, from self-subsistence to commercial cash cropping) on chemical exposures and on the well-being of people [19]. The study communities were selected so that they varied in terms of their subsistence patterns. The study sites included five communities within the Citarum Watershed, West Java, Indonesia: Bongas (B), an agricultural village facing the Saguling dam with a fish culture and rice cropping; Cihawuk (C), an agricultural village with vegetable cropping; Taruma Jaya (T), an agricultural village with dairy husbandry and vegetable cropping; Pasir Pogor (P), an agricultural village with rice cropping; and Sekeloa (S), an urban community in Bandung city (Figure 1).

Data collection was conducted from August to September 2006 in communities B, C, and T, and in March 2007 in communities P and S. One of the authors (Budhi Gunawan) selected two elementary schools from each community and obtained the permission from the school head to collaborate on this project. Then, school teachers selected 50 students in the 5th and 6th grades of each elementary school who met the inclusion criteria. The inclusion criteria for a student were that his/her mother, father, and sister/brother could participate in the study. In case we could not obtain 50 students from the 5th and 6th grades of each elementary school, students whose mothers or fathers were not available but another adult (aged 20 years or above) was available were recruited for the study. These selection criteria were used to enable us to examine within (siblings or husband and wife) and between household variation in terms of chemical exposures and their related health effects. In each household that fulfilled the selection criteria, four members (two adults and two children) were invited to participate in the study. A total of 929 people participated in the ENVRERA project in the five communities. Because the ages of the sisters/brothers of 5th or 6th grade school children were diverse, this paper only targeted 5th or 6th grade school children. Furthermore, as our interest was to analyze double burden for mother-child and father-child pairs and it was difficult to recruit fathers to a survey conducted during the daytime, this paper only targets 242 children in 5th or 6th grade whose mothers were also available. Thus, the final sample for this paper was 242 children and their mothers (*n* = 242) and fathers (*n* = 225). Breakdown of the number of participants by community was as follows; 242 children (communities B = 51, C = 52, T = 4 8, P = 50, S = 41), 242 mothers (communities B = 51, C = 52, T = 48, P = 50, S = 41), and 225 fathers (communities B = 47, C = 52, T = 48, P = 49, S = 29).

### 2.2. Physical Check-up

A health camp was set up at each elementary school where questionnaire surveys on socioeconomic characteristics, anthropometric measurements, urine and blood sampling and testing, and questionnaire surveys on their food consumption habits using food frequency questionnaires (FFQ) were conducted.

### 2.3. Questionnaire Survey on Socioeconomic Characteristics

Names, ages, genders, occupations, and education history of all household members, as well as possession of goods of the household, water source and sanitation, and land ownership were asked using a structured questionnaire. Possession of goods was asked for five items including a radio, TV, refrigerator, telephone, and mobile phone. Possession of each item was scored as 1, and a total score was used as a “possession of goods” score.

### 2.4. Anthropometric Measurements

Anthropometric measurements were taken by one of the authors (Makiko Sekiyama) following standard methods [20]. Body weight was measured to the nearest 0.1 kg and the percentage of body fat (BF%) was estimated using bioelectrical impedance with a body composition analyzer (DC-320, Tanita Co., Ltd., Tokyo, Japan). Height was measured to the nearest 0.1 cm using a portable stadiometer. Skinfold thicknesses at the biceps, triceps, subscapular, and suprailiac were measured three times with GPM skinfold calipers (Siber Hegner & Co., Ltd., Zurich, Switzerland) that can measure up to 40 mm with a precision of 0.2 mm. The three measurements at each site were averaged for the statistical analyses. For an adult, using skinfold thickness data, BF% was calculated according to Durnin and Womersley [21]. Waist and hip circumference was measured with a plastic tape with a precision of 1 mm and waist-to-hip ratio (WHR) was calculated for adults. Mid-upper arm circumference (MUAC) was also measured with a plastic tape with a precision of 1 mm. For children, z-scores for height-for-age (HAZ) were calculated as nutritional indicators based on the WHO growth references published in 2007 for children older than 5 years [22] using EPI-INFO (Version 7, Centers for Disease Control and Prevention, Atlanta, GA, USA).

### 2.5. Hemoglobin (Hb)

Capillary whole blood was collected via finger prick from each participant by one of the authors (Linda Dewanti; an Indonesian physician), with Hb concentrations measured on site using a battery operated photometric analyzer (Test-mate; EQM Research, Cincinnati, OH, USA).

### 2.6. FFQ (Food Frequency Questionnaire)

A FFQ including 22 food items was developed based on the results of one of the author’s (Makiko Sekiyama) preliminary surveys [23,24]. The 22 food items were rice, potato, tofu/tempeh, fresh vegetable, cooked vegetable, indigenous fruit, non-native fruit, egg, salted fish, freshwater fish, sea fish, chicken, beef, goat meat, duck meat, noodle, tea/coffee, milk, meatball, fried sweets, bread, and snack. The consumption frequency was asked using 9 alternatives and converted into weighing factors for statistical analysis: (1) almost never = 0; (2) one to three times per month = 0.07; (3) once per week = 0.1; (4) two to four times per week = 0.4; (5) five to six times per week = 0.8; (6) once per day = 1; (7) two to three times per day = 2.5; (8) four to six times per day = 5; and (9) more than six times per day = 6. Indigenous fruits are those planted locally such as papayas and guavas, whereas non-native fruits are those not planted locally but available in the market or local shop such as apple, orange, and grape.

### 2.7. Ethics

Ethical approval for the study was obtained from the Research Ethics Committee at Graduate School of Medicine, the University of Tokyo (Approval No. 1505) and Padjadjaran University. The purpose and procedures of the study were explained to the participants and written informed consent was obtained from all study subjects.

### 2.8. Statistical Analysis

Stunting was defined as a height-for-age z-score (HAZ) <−2 according to the World Health Organization (WHO) growth standards [22]. BMI was calculated as a ratio of weight (kg)/height (m)^2^. Concerning the overweight cutoff, Asian populations were found to have a higher level of BF% at lower levels of BMI than other ethnic groups [25]. Thus, adult overweight was classified as a BMI (in kg/m^2^) ≥23 to capture the increased risk of NCDs [26]. Double burden was defined as coexistence of maternal or paternal overweight and child stunting within the same household. Namely, paternal-child double burden was coexistence of paternal overweight and child stunting within the same household, and maternal-child double burden was coexistence of maternal overweight and child stunting within the same household.

A principal component analysis based on the 22 food items was conducted to assess the major dietary patterns among the subjects. In determining the number of factors to retain, we considered the results of the Scree test, eigenvalues greater than 1, and interpretability of the factors [27]. Factors were then rotated with an orthogonal rotation procedure (varimax rotation). Labeling of dietary patterns was based on the interpretation of foods with high factor loadings for each dietary pattern [28]. Only foods with a factor loading ≥|0.25| were included in this study. Factor scores for each dietary pattern were categorized into quartiles (quartile 1 represented a low intake of the food pattern; quartile 4 represented a high intake of the food pattern), separately for child, mother, and father. Association between dietary patterns with child stunting, with maternal overweight, and with paternal overweight was analyzed using logistic regression analysis, adjusted for potential confounders.

Age, gender, and physical characteristics were compared between maternal-child double burden and non-double burden households. Comparisons between double burden and non-double burden households were conducted using a *t*-test for maternal height, which was the only variable to fit a normal distribution. For other parameters that did not fit a normal distribution, comparisons were made using the Mann-Whitney *U*-test. Relevant socioeconomic factors associated with maternal-child double burden were analyzed using logistic regression analyses. Descriptive statistics were used to examine the full distribution of variables. Using appropriate cutoffs, categorical variables were created for maternal age (<30, 30–39.9, or ≥40 year), maternal education (no schooling, elementary or secondary school, or >secondary school), maternal height (<145, 145–149.9, or ≥150 cm), and parity (1–2, 3–5, or ≥6). Each socioeconomic factor was firstly put in the univariate logistic regression analysis to examine its association with maternal-child double burden, then only those associated at the *p* < 0.1 level were included in the multiple logistic regression models. Associations between dietary patterns of mother and child and the maternal-child double burden were also analyzed using logistic regression analysis, adjusted for potential confounders. For all analyses using logistic regression models, odds ratios (ORs) and corresponding 95% confidence intervals (CIs) were calculated with statistical significance defined as *p* < 0.1. All analyses were performed using the Statistical Package for Social Science (SPSS) software package (Version 10.0, SPSS Inc., Chicago, IL, USA).

## 3. Results

### 3.1. Characteristics of the Subjects

Table 1 shows the ages and physical characteristics of the study subjects. For the children, the mean age was 11.1 years, mean HAZ was −2.15, and the stunting ratio was 57.9%. For the fathers, the mean age was 41.6 years, mean BMI was 21.1 kg/m^2^, and the proportion of overweight (BMI ≥ 23) was 18.7%. For the mothers, the mean age was 36.9 years, mean BMI was 23.8 kg/m^2^, and the proportion of overweight (BMI ≥ 23) was 53.7%.

**Table 1:** Characteristics of child, father, and mother subjects.

	*n*	Age	Height (cm)	HAZ ^a^	Stunting (HAZ < −2)	Weight (kg)	BMI ^b^ (kg/m^2^)	Overweight (BMI ≥ 23)
		Mean ± SD ^c^	Mean ± SD	Mean ± SD	%	Mean ± SD	Mean ± SD	%
Child	242	11.1 ± 0.86	132.8 ± 7.08	–2.15 ± 1.01	57.9	27.9 ± 5.33	15.7 ± 1.73	
Father	225	41.6 ± 9.06	160.1 ± 6.35			54.1 ± 8.43	21.1 ± 2.68	18.7
Mother	242	36.9 ± 6.83	149.1 ± 4.97			53.1 ± 9.15	23.8 ± 3.67	53.7

^a^ Height-for-age z-score; ^b^ Body mass index; ^c^ Standard deviation.

Socioeconomic characteristics of the subject households were also obtained from the questionnaire survey. Regarding the land ownership, only 13.2% of the households owned paddy fields and 22.4% of the households owned vegetable fields. Consequently, though the main occupation of the subject father was agricultural work (30.2%), 60.2% of them were agricultural wage laborers. The main water source of the subject households were spring (60.8%) and well (37.3%), while some of the households in community B used lake water especially in dry season due to the scarcity of well water. 57.2% of the subject households owned their private toilet facilities, whereas 49.7% of them used the shared or public toilet facilities.

With regard to the birth-related factors, the mean parity of the subject mothers was 3.28 ± 1.63. Information of birth weight, which is the important birth-related factors affecting future nutritional status, was not obtained from the study subjects, because the bulk of child deliveries were made at home without measurement of birth weight.

### 3.2. Paternal-Child and Maternal-Child Double Burden

Table 2 shows that paternal-child double burden (coexistence of paternal overweight and child stunting) was found only in 8.4% of the targeted households, whereas maternal-child double burden (coexistence of maternal overweight and child stunting) was observed in 30.6% of the targeted households.

**Table 2:** Maternal-child and paternal-child pair double burden.

		Non-Obese (%)		Obese (%)	
		Father	Mother	Father	Mother
**Child**	**Non-Stunting (%)**	29.8	19.0	10.2	23.1
	**Stunting (%)**	51.6	27.3	8.4	30.6

### 3.3. Dietary Patterns of the Subjects

Two major dietary patterns, explaining 25.7% of the total variance in the consumption of the 22 food items, were identified in the principal component analysis. For each of the two major dietary patterns, foods with a high factor loading (set at 0.25 or greater) are shown in Table 3. The first dietary pattern was named as the “Modern” dietary pattern, which was characterized by a high consumption of flesh foods such as egg (factor loading = 0.559), freshwater fish (0.557), and milk (0.524), and of instant snack foods such as meatball (0.541), fried sweets (0.483), noodle (0.473), and snack (0.462). The second dietary pattern was named as the “High-animal products” dietary pattern because it was characterized by high consumption of animal products such as beef (0.556), goat (0.487), chicken (0.349), and duck (0.258), but low consumption of salted fish (−0.465). One example of the salted fish available in this area is the small fish of the *Engraulidae* family (called *ikan teri* in the Indonesian language). Because of its salty taste, local people consume very small portions of salted fish with large amounts of rice.

**Table 3:** Factor loading matrix for the major factors (dietary patterns) identified by using food consumption data.

	Factor 1 (“Modern” Dietary Pattern)	Factor 2 (“High-Animal Products” Dietary Pattern)
Rice		
Potato	0.322	
Tofu/tempeh	0.543	
Fresh vegetable		–0.406
Cooked vegetable		–0.340
Indigenous fruit	0.624	
Non-native fruit	0.420	0.593
Egg	0.559	
Salted fish		–0.465
Freshwater fish	0.557	
Sea fish	0.387	
Chicken	0.475	0.349
Beef	0.333	0.556
Goat		0.487
Duck		0.258
Noodle	0.473	
Tea/coffee		–0.397
Milk	0.524	0.261
Meatball	0.541	
Fried sweets	0.483	–0.296
Bread	0.429	
Snack	0.462	

Absolute values < 0.25 were not listed in the table; the first factor explained 16.7% of the total variance and the second factor explained 8.96% of the total variance.

### 3.4. Dietary Patterns and Child Stunting, Paternal Overweight, and Maternal Overweight

Table 4 shows associations between dietary patterns of children and child stunting, dietary patterns of fathers and paternal overweight, and dietary patterns of mothers and maternal overweight. After controlling for potential confounding factors, mothers in the middle (Q2–Q3) and highest quartile (Q4) of the “Modern” dietary pattern had higher risk of overweight (Adjusted OR = 2.34, 95% CI = 1.20–4.57 for Q2–Q3; Adjusted OR = 2.63, 95% CI = 1.12–6.17 for Q4) compared with the lowest quartile (Q1 = reference). Children in the highest quartile (Q4) of the “High-animal products” dietary pattern had a lower risk of stunting (Adjusted OR = 0.36, 95% CI = 0.16–0.80) than those in the lowest quartile (Q1). Fathers in the highest quartile (Q4) of the “High-animal products” dietary pattern had a higher risk of overweight (Adjusted OR = 3.92, 95% CI = 1.13–13.6) than those in the lowest quartile (Q1). Confounding factors considered in this analysis were age, gender, and possession of goods for children, and age, possession of goods, and occupation for fathers and mothers. Possession of goods was used as a proxy for household socioeconomic status and occupation was used as a proxy for physical activity level.

**Table 4:** Association of the two identified dietary patterns with child stunting, maternal overweight, and paternal overweight.

			Child Stunting		Maternal Overweight		Paternal Overweight	
			OR (95% CI)	*p*	OR (95% CI)	*p*	OR (95% CI)	*p*
Model 1 ^a,c^	Modern	Q1	1.00	–	1.00	–	1.00	–
		Q2–Q3	0.85 (0.45–1.61)	0.623	2.60 (1.36–4.98)	0.004	1.81 (0.68–4.80)	0.232
		Q4	0.76 (0.37–1.57)	0.459	3.53 (1.65–7.53)	0.001	3.33 (1.19–9.30)	0.021
	High-animal products	Q1	1.00	–	1.00	–	1.00	–
		Q2–Q3	0.59 (0.30–1.14)	0.115	0.89 (0.48–1.66)	0.708	2.83 (0.92–8.71)	0.071
		Q4	0.38 (0.18–0.79)	0.010	1.07 (0.52–2.21)	0.853	6.16 (1.93–19.7)	0.002
Model 2 ^b,c^	Modern	Q1	1.00	–	1.00	–	1.00	–
		Q2–Q3	1.03 (0.53–2.00)	0.929	2.34 (1.20–4.57)	0.013	1.28 (0.45–3.60)	0.647
		Q4	1.01 (0.46–2.21)	0.980	2.63 (1.12–6.17)	0.026	1.00 (0.30–3.26)	0.994
	High-animal products	Q1	1.00	–	1.00	–	1.00	–
		Q2–Q3	0.60 (0.30–1.19)	0.143	0.92 (0.48–1.76)	0.802	2.59 (0.78–8.54)	0.119
		Q4	0.36 (0.16–0.80)	0.012	0.95 (0.44–2.04)	0.892	3.92 (1.13–13.6)	0.032

^a^ Unadjusted; ^b^ Adjusted for age, gender, and possession of goods for children; adjusted for age, possession of goods, and occupation for fathers and mothers; ^c^ For each dietary pattern, quartile values were separately calculated for child, mother, and father; associations between child dietary patterns and child stunting, between maternal dietary patterns and maternal overweight, and between paternal dietary patterns and paternal overweight were examined.

### 3.5. Age, Gender, and Physical Characteristics of Maternal-Child Double Burden Households

Age, gender, and physical characteristics were compared between maternal-child double burden and maternal-child non-double burden households (Table 5). For the children, age and gender were not different between double burden and non-double burden groups. As for physical characteristics of the children, height, weight, HAZ, BMI, waist, hip, MUAC, sum of skinfold thickness, and Hb were compared between double burden and non-double burden groups. In terms of physical characteristics of the mothers, height, weight, BMI, waist, hip, WHR, MUAC, sum of skinfold thickness, BF% measured by bioelectrical impedance (BF%-BI), BF% calculated using skinfold thickness based on Durnin and Womersley’s (1974) formula (BF%-ST), and Hb were compared between double burden and non-double burden groups. The results show that for the children, height (*p* < 0.001) and HAZ (*p* < 0.001) were significantly lower in the double burden group. For mothers, weight (*p* < 0.001), BMI (*p* < 0.001), waist (*p* < 0.001), hip (*p* < 0.001), WHR (*p* < 0.05), MUAC (*p* < 0.001), sum of skinfold thickness (*p* < 0.001), BF%-BI (*p* < 0.001), BF%-ST (*p* < 0.001), and Hb (*p* < 0.01) were significantly higher in the double burden group. Mothers from double burden households showed high adiposity: BF%-BI = 39.0% ± 5.51%, BF%-ST = 35.1% ± 6.90%, waist = 82.1 ± 9.38 cm.

### 3.6. Association of Maternal-Child Double Burden with Sociodemographic Variables

Table 6 shows sociodemographic characteristics of maternal-child double burden and maternal-child non-double burden households and the association with maternal-child double burden. Sociodemographic factors that were significantly associated with maternal-child double burden included age of the child (OR = 1.34, 95% CI = 0.96–1.88), maternal education higher than secondary school level (OR = 0.33, 95% CI = 0.09–1.15), and maternal occupation as housewife (OR = 0.49, 95% CI = 0.22–1.12) in the univariate analysis. Then, the variables that were significantly associated with maternal-child double burden in the univariate analysis (*p* < 0.1) were entered into a multivariate analysis. The results show that age of the child (Adjusted OR = 1.44, 95% CI = 1.00–2.08) and maternal occupation as housewife (Adjusted OR = 0.45, 95% CI = 0.19–1.05) remained as significant factors for maternal-child double burden.

### 3.7. Association of Maternal-Child Double Burden with Dietary Patterns of Mother and Child

Table 7 shows associations between dietary patterns of child and mother, and maternal-child double burden from the logistic regression analysis. After controlling for the confounding factors detected in Table 6, children in the highest quartile of the “High-animal products” dietary pattern had a lower risk of maternal-child double burden (Adjusted OR = 0.46, 95% CI = 0.21–1.04) than those in the lowest quartile.

**Table 5:** Age, gender, and physical characteristics of children and mothers from maternal-child double burden and maternal-child non-double burden households.

	Double Burden	*n*	Non-Double Burden	*n*	*p* ^b^
	Mean ± SD ^a^ (*N* = 73)		Mean ± SD (*N* = 169)		
Child characteristics					
Age in months	140.5 ± 8.94		138.0 ± 10.8		0.154
Gender					
Male		32		83	0.834
Female		41		86	
Height (cm)	129.8 ± 4.64		134.2 ± 7.54		0.000
Weight (kg)	26.7 ± 3.40		28.5 ± 5.90		0.055
HAZ ^c^	−2.72 ± 0.53		−1.90 ± 1.06		0.000
BMI	15.8 ± 1.40		15.7 ± 1.86		0.217
Waist (cm)	56.6 ± 3.91		57.5 ± 5.74		0.364
Hip (cm)	69.1 ± 6.18		69.8 ± 5.96		0.264
MUAC (mm)	18.4 ± 1.44		19.0 ± 4.84		0.771
Sum of skinfold thickness (mm)	31.5 ± 7.34		31.8 ± 11.7		0.321
Hb (g/dL)	12.7 ± 1.55		13.1 ± 1.00		0.184
Maternal characteristics					
Age	35.7 ± 6.20		37.4 ± 7.03		0.074
Height (cm)	148.7 ± 5.25		149.3 ± 4.85		0.620
Weight (kg)	58.2 ± 6.87		50.9 ± 9.14		0.000
BMI ^d^ (kg/m^2^)	26.3 ± 2.29		22.8 ± 3.66		0.000
Waist (cm)	82.1 ± 9.38		75.5 ± 13.0		0.000
Hip (cm)	97.9 ± 10.4		91.8 ± 13.5		0.000
WHR ^e^	0.86 ± 0.23		0.82 ± 0.16		0.016
MUAC ^f^ (mm)	28.2 ± 2.06		26.2 ± 5.87		0.000
Sum of skinfold thickness (mm)	86.5 ± 23.9		64.0 ± 28.6		0.000
BF%-BI ^g^ (%)	39.0 ± 5.51		32.9 ± 6.61		0.000
BF%-ST ^h^ (%)	35.1 ± 6.90		31.7 ± 6.80		0.000
Hb ^i^ (g/dL)	13.4 ± 1.30		12.6 ± 1.84		0.001

^a^ Standard deviation; ^b^ Comparisons between double burden and non-double burden households were conducted using a *t*-test for maternal height, which was the only variable to fit a normal distribution; for other parameters, which did not fit a normal distribution, comparisons were made using the Mann-Whitney U-test; ^c^ Height-for-age z-score; ^d^ Body mass index; ^e^ Waist to hip ratio; ^f^ Mid-upper arm circumference; ^g^ Body fat percentage measured by bioelectrical impedance; ^h^ Body fat percentage calculated using skinfold thickness based on Durnin and Womersley’s (1974) formula [21]; ^i^ hemoglobin.

**Table 6:** Socioeconomic characteristics and associations with maternal-child double burden.

	Double Burden		Non-Double Burden		Univariate		Multivariate ^a^	
	%	Mean ± SD	%	Mean ± SD	OR (95% CI)	*p*	Adjusted OR (95% CI)	*p*
Child characteristics								
Age		11.2 ± 0.74		11.0 ± 0.90	1.34 (0.96–1.88)	0.089	1.44 (1.00–2.08)	0.051
Gender								
Male	43.8		49.1		1.00 (Reference)	–		
Female	56.2		50.9		1.24 (0.71–2.15)	0.451		
Maternal characteristics								
Age								
<30 year	15.5		12.7		1.00 (Reference)	–		
30–40 year	54.9		44.6		1.01 (0.44–2.30)	0.988		
≥40 year	29.6		42.8		0.57 (0.24–1.36)	0.202		
Education								
No schooling/Elementary school	23.6		51.9		1.00 (Reference)	–	1.00 (Reference)	–
Secondary school	5.1		9.7		1.15 (0.53–2.47)	0.727	1.29 (0.59–2.82)	0.526
>Secondary school	1.3		8.4		0.33 (0.09–1.15)	0.083	0.35 (0.10–1.27)	0.111
Height								
<145 cm	22.5		15		1.00 (Reference)	–		
145–149.9 cm	32.4		38.9		0.55 (0.25–1.22)	0.14		
≥150 cm	45.1		46.1		0.65 (0.31–1.38)	0.26		
Parity								
1–2	9		27.8		1.00 (Reference)	–		
3–5	18.4		35		1.62 (0.88–3.00)	0.123		
≥6	3		6.8		1.35 (0.49–3.74)	0.558		
Occupation								
Farmer	5.1		6.3		1.00 (Reference)	–	1.00 (Reference)	–
Merchant, services	2.1		5.1		0.52 (0.14–1.89)	0.322	0.59 (0.15–2.24)	0.435
Other wage labor	0		0.8		0.00 (0.00–0.00)	0.999	0.00 (0.00–0.00)	0.999
Housewife	22.8		57.8		0.49 (0.22–1.12)	0.091	0.45 (0.19–1.05)	0.064
Household characteristics								
Possession of goods		3.53 ± 1.74		3.59 ± 1.82	0.98 (0.84–1.15)	0.819		

^a^ The variables that were associated with maternal-child double burden in the univariate analysis (*p* < 0.1) were entered into multivariate analysis; SD: Standard deviation; OR: odds ratio; CI: confidence interval.

**Table 7:** Association of double burden with dietary patterns.

			Child Diet		Maternal Diet	
			OR (95% CI)	*p*	OR (95% CI)	*p*
Model 1 ^a^	Modern	Q1	1.00	–	1.00	–
		Q2–Q3	1.07 (0.55–2.09)	0.847	0.99 (0.50–1.95)	0.972
		Q4	0.92 (0.42–2.03)	0.841	0.92 (0.42–2.03)	0.840
	High-animal products	Q1	1.00	–	1.00	–
		Q2–Q3	0.79 (0.41–1.51)	0.479	1.36 (0.68–2.71)	0.381
		Q4	0.48 (0.21–1.07)	0.073	0.92 (0.40–2.08)	0.834
Model 2 ^b^	Modern	Q1	1.00	–	1.00	–
		Q2–Q3	1.12 (0.57–2.21)	0.740	1.01 (0.51–2.02)	0.972
		Q4	0.98 (0.44–2.16)	0.949	0.92 (0.40–2.11)	0.839
	High-animal products	Q1	1.00	–	1.00	–
		Q2–Q3	0.81 (0.42–1.57)	0.786	1.45 (0.72–2.95)	0.300
		Q4	0.46 (0.21–1.04)	0.064	1.01 (0.43–2.33)	0.991

^a^ Unadjusted; ^b^ Adjusted for child’s age and mother’s occupation, which were significantly associated with maternal-child double burden (Table 6); OR: odds ratio; CI: confidence interval.

## 4. Discussion

### 4.1. Double Burden Structure at the Household Level

To the best of our knowledge, this is the first study that examined double burden structure at the household level for both mother-child and father-child pairs in the same household using primary data in Indonesia. We found that double burden in mother-child pairs exist in 30.6% of the subject households, whereas that of father-child pairs exists in only 8.4% of the subject households.

The percentage of double burden in mother-child pairs observed in this study was higher than that of the previous study conducted in rural Indonesia [6]. Those authors reported that maternal overweight and child stunting coexisted in 11% of the rural population throughout Indonesia. As pointed out by Vaezghasemi [18], the prevalence of double burden largely differs across the provinces in Indonesia. Among 13 provinces analyzed in Vaezghasemi’s study, the prevalence of double burden in West Java was 4th highest. Thus, the higher prevalence of double burden observed in maternal-child pairs in our study would be partly attributable to this regional difference.

Our study revealed that the risk of double burden within the same household was larger among mother-child pairs than for father-child pairs. Among households whose mothers were overweight, only 24.0% had an overweight father and the rest (76.0%) had a non-overweight father. This gender difference in the prevalence of overweight has been reported in several studies in Indonesia [2,29], and is more pronounced than other countries in Asia such as China, Vietnam, and Nepal [30].

### 4.2. Adiposity Related Physical Characteristics of Double Burden Household Members

A novel aspect of our study is that the data include physical measurements related to adiposity. Among children, physical characteristics were not different between double burden and non-double burden groups except for height and HAZ. Among mothers, however, almost all physical characteristics differed between the double burden and non-double burden groups. Mothers in the double burden group had higher BF%, waist and hip circumference, WHR, MUAC, and the sum of skinfold thickness than those in the non-double burden group.

It is frequently stated in the literature that in the case of obesity or adiposity the BF% exceeds 25% in males and 35% in females [12,31]. In our analyses, we used two methods, BF%-BI and BF%-ST, for estimating BF%. The percentage of mothers whose BF% exceeded 35% was 87.3% using the BF%-BI method and was 66.2% using the BF%-ST method. A Tanita bioelectrical impedance with a body composition analyzer is often reported to underestimate BF% especially for fat individuals [32]. Durnin and Womersley’s [21] formula has been used to estimate BF% in Indonesian adults [33,34,35] and has been reported to underestimate BF% by 1% compared with the deuterium dilution technique [35]. Considering these technical biases, it was judged that at least 66% of mothers from double burden households have obesity or adiposity based on BF% criteria. Moreover, 60.6% of mothers from double burden households had a waist larger than 80 cm, which is frequently used as an Asian threshold of waist circumference for metabolic syndrome [36]. It has been noted for a given BMI that Asians have a higher body fat percentage compared with Caucasians [25], and thus we determined overweight as a BMI (in kg/m^2^) ≥23 to capture the increased risk of NCDs [26]. Even with this strict definition of overweight, more than 60% of mothers from double burden households were categorized either with adiposity or metabolic syndrome.

### 4.3. Dietary Patterns Relevant to Double Burden

Studies on the double burden problem in Indonesia scarcely mentioned the association between double burden and dietary patterns. In this study, to identify the dietary patterns of the study subjects, we administered a FFQ not at the household level but at the individual level, though we recruited father, mother and child pairs from the same household. In the subject area, frequent snacking outside the house is commonly observed not only for adults but also for children [23]. Also, family members do not always take their meals together. Thus, it was judged to be necessary to obtain FFQ data from each individual. Using the FFQ data, dietary patterns of the study subjects were identified by means of principal component analysis and two major dietary patterns, “Modern” and “High-animal products”, were identified.

The “Modern” dietary pattern was predominantly characterized by higher consumption of flesh foods and instant snack foods. In this study area, instant snack foods such as meatball (called *bakso* in the Indonesian language), noodle, and fried sweets are sold in retailers in the village (called *Warung* in the Indonesian language) and peddlers. People often consume these snack foods instead of taking their meals and the extent of the contribution of these foods to overall nutrition is relatively high fat but low micronutrients [23]. A considerable body of literature has reported that traditional population groups throughout the world are replacing their traditional food patterns rich in complex carbohydrates, micronutrients, and fiber with diets high in refined sugars, animal products, and highly processed foods [37]. Considering the contents and the greatest preference in the principal component analysis, this dietary pattern likely reflects the nutrition transition phenomenon in this subject area. With regards to the relationships with nutritional status, after controlling for confounding factors, mothers in the middle quartiles and the highest quartile of the “Modern” dietary pattern had a higher risk of overweight. This finding is in agreement with other studies that reported that modified dietary patterns after the initiation of nutrition transition, frequently called a “Western diet”, have a positive association with overweight among women [38,39].

The second dietary pattern identified by principal component analysis was the “High-animal products” dietary pattern, which was characterized by high consumption of animal products such as beef, goat, chicken, and duck but low consumption of salted fish. With regards to its association with nutritional status, after controlling for confounding factors, children in the highest quartile had a lower risk of stunting than those in the lowest, and fathers in the highest quartile had a higher risk of overweight than those in the lowest. In developing countries, consumption of animal products such as meat is often low in rural areas because of economic constrains [40]. Our data show that the median intake frequency of meat was mostly less than once per week among the subjects in the lowest quartile of the “High-animal products” dietary pattern; for example, among children, median intakes of chicken: one to three times per month; beef: almost never; goat: almost never; and duck: almost never. Animal products such as meat are not only the source of the animal proteins but also readily available sources of iron, zinc, and preformed vitamin A [40]. It has been reported in many studies that deficient intake of iron, zinc, and vitamin A, which are serious problems in some places in the world, impairs linear growth in children [41,42].

In our analysis, the “High-animal products” dietary pattern was associated with decreased risk of maternal-child double burden; after controlling for confounding factors, children in the highest quartile had a lower risk than those in the lowest. This association was highly related to the strong negative correlation of the “High-animal products” dietary pattern with child stunting. There was no association between the “High-animal products” dietary pattern and maternal overweight. For example, quartiles of the “High-animal products” dietary pattern were not different between overweight and non-overweight mothers (by χ^2^ test), but quartiles of the “High-animal products” dietary pattern among overweight mothers were significantly different between those with stunted children and those with non-stunted children (*p* < 0.1 by χ^2^ test). Thus, it was judged that it is critical to improve the issue of child stunting through adequate intake of animal products.

### 4.4. Limitations

Our study has a few limitations that should be considered. First, the sample size is small compared with previous studies discussing double burden issues in Indonesia. However, it is difficult to recruit father- and mother-child pairs from the same household to understand double burden structure in the household. Further, adiposity related physical characteristics and detailed information of food consumption frequencies are not easy to assess when targeting a large sample. Second, we adapted cutoff points for Asian populations for the classification of overweight, taking into account that Asians tend to have higher risk of NCDs at lower BMIs. This means that caution is warranted in terms of comparing our results with previous studies. Third, our food consumption data do not offer quantitative data and, thus, principal components analysis was conducted based on the consumption frequency. Even with this limitation, we adapted the principal components analysis considering the increasing interest in nutritional epidemiology to capture the association between diet and its health effects through dietary patterns, not through a single nutrient or food, because combined effects and interactions of multiple nutrients cannot be captured by analyzing a single nutrient or food [27,38,43]. Despite these limitations, we believe the present study provides useful information for understanding the degree of emerging household-level double burden of malnutrition in Indonesia.

## 5. Conclusions

The prevalence of double burden of malnutrition within the same household was three to four times higher for mother-child pairs than for father-child pairs in West Java, Indonesia. Furthermore, mothers from double burden households showed high levels of adiposity. Thus, further studies and policies should address overweight and the risk of NCDs among overweight mothers in double burden households as a rising health threat in Indonesia. This study also demonstrated the significance of dietary pattern, which has been scarcely examined before in studies on double burden of malnutrition. Dietary pattern characterized by high consumption of animal products but low consumption of salted fish, as identified by a principal component analysis, was associated with the decreased risk of maternal-child double burden through a strong negative correlation with child stunting. Therefore, improving child stunting through adequate intake of animal products is critical to solve the problem of maternal-child double burden in Indonesia.
